# Laughing Gas

**Published:** 1864-01

**Authors:** A. Westcott

**Affiliations:** Syracuse


Laughing Gas.—The following is from the Syracuse Journal, written by Dr. A. Wescott, in which he discusses Dr. Colton's views pretty closely; yet we doubt whether the presentation of the theory made by Dr. A. will be fully sustained in all particulars by experience, at least so it seems by the experience thus far attained.—Ed. Reg.

Not long since, several lectures and exhibitions were given at Wieting Hall, by an itinerant lecturer, by the name of Collins, upon this so-called laughing gas.

During their progress, an article as *editorial*, but evidently prepared by Collins himself, appeared in two of our daily papers—and as this publication, and the lectures to which it alludes, seem to have had a wonderful effect upon this community, I propose as briefly as possible to analize the merits of this gas as an anæsthetic agent.

The editorial is as follows:

"The 'Laughing Gas.'—By invitation quite a number of gentlemen met at Wieting Hall on Saturday evening to witness a private exhibition of this wonderful gas. Laughing gas, as many of our readers are probably aware, is known in scientific phrase as nitrous oxyd. *It consists merely of the elements of the common air*—nitrogen and oxygen—*mixed in a little different proportions.* It has a larger proportion of the life-giving principle, oxygen, than the atmosphere, and *hence* when inhaled makes one *live much faster* than when he breathes the common air.

"The blood is more rapidly purified in the lungs, rushes more tumultuously through the arteries, quickens the pulse, mounts to the brain and plays riot in the airy chambers of the mind. The individual becomes exhilerated and careless of observation, throws out in word and act whatever is uppermost in the mind. The effect is very pleasing but transitory, and we learn that *there is no possible danger of a person doing or saying anything which he might have occasion to regret.*

"On Saturday evening we were treated to snatches of songs, bits of oratory, political harangues and capital dancing. If the audience are as much amused to-night as those present on Saturday evening, the public will call upon Mr. Collins for a repetition of this *laughing entertainment.*"

Now had this speculator in the health and lives of our citizens written this article expressly to show his profound ignorance, not only of the human system, but of every branch of medicine and chemistry, he could not have been more successful; and yet, strange to say, by just such articles as these, backed by a flood of similar *advertisements*, the public are led, nay constrained to believe that this gas is not only always efficient in producing the desired effect, but that it is *always harmless.* From an advertisement now before me, a dentist of Bond street, New York, and one who claims to be at the head of a "*Dental Association*" *bearing his own name*, gives the following *five reasons* why all should use it who wish to become temporarily insensible to pain, to-wit:

1st. It is perfectly harmless.
2d. The insensibility is perfect.
3d. It is pleasant to inhale.
4th. The whole time occupied in the inhalation and extracting from three to ten teeth, and returning to *perfect* consciousness, does not exceed *three minutes.*
5th. *It can be given with safety in all sorts and stages of disease!!!*

Now what rational person can, for one moment, swallow such a pill as is here presented—sugar-coated though it be? Who can suppose that any *sound* person, not to say those in the last stages of disease, can be brought into so unnatural a state in "*thirty seconds*" *perfectly harmlessly?*

The two important questions as regards the use of this gas for dental purposes, are these, to-wit:

1st. Will it "*always*," or, indeed, generally produce the desired effect, or *insensibility to pain?*
2d. Is it harmless, even in the most judicious hands?

As regards the former question, I have to confess that I have no experience with it "for dental purposes," but having been engaged for six consecutive years, at one period of my life, in practical chemistry, I have administered it from one to two hundred times as a scientific experiment, and I can say that in no instance did I ever see a person under its influence in a fit state to have teeth extracted, or any other surgical operation performed, or even where teeth could be extracted without danger to the *operator*, if not the patient.

In these experiments it was given only for its exhilarating effects, and *never* designedly carried (for reasons which we then supposed to be good, and which I shall allude to hereafter), to the point of stupefying the person inhaling it.

This Mr. Collins asserts, among other things, in his editorial, that "there is no possible danger of a person doing or saying anything they might have occasion to regret." This is by no means my own experience with this gas. I have often witnessed the most pious using the most profane, and the delicate the most obscene language; and the most fastidious persons are liable to cut antics the mention of which would make them blush when rational. But this is by no means the great object of its use.

The position which I shall take and endeavor to establish by facts and fair deductions is, that it is always dangerous,—not that it necessarily proves hurtful in all cases, but that it would require superhuman skill to tell whether the subject might or might not have some organic defect or disease. But a very short time since, a soldier died suddenly in this city, who had just previously been examined by a medical board and pronounced sound and "able-bodied." So sudden and mysterious was his death, that the woman with whom he was at the time boarding was arrested and imprisoned for his murder. But a post-mortem examination, however, released this woman from prison and relieved the public of all doubt as to the cause of his death. "One lung was found to be entirely destroyed by disease, and the other was nearly consumed!" Now if such mistakes as these were liable to be committed by a board of medical men, entrusted with the responsible duty of selecting fit and able-bodied men for our army, what will be the chances for similar mistakes, by such men as are urging this gas upon the community, as "perfectly harmless,"—not one in ten of which are medically educated or in anyway qualified to make such examination?

But, before offering any direct testimony upon this point, let us examine for a moment the logic of this Mr. Collins, who seems to have been the oracle and file-leader of this region, and who for a number of evenings drew large audiences at Wieting Hall, as a *lecturer* upon the *sciences* of this gas. The general ideas put forth in these lectures are rehearsed in the "editorial" above-quoted. His explanation of its *harmlessness* and *exhilarating qualities* are thus stated by him:

"It consists *merely of the elements of the common air—nitrogen and oxygen, mixed in a little different proportions. It has a larger proportion of the life giving principle—oxygen, than the atmosphere; and hence, when inhaled, makes one live much faster than when he breathes common air!*"

In this attempted explanation, the writer has shown himself wholly ignorant of the laws of chemical affinity. He has blended a *mixture* with a *chemical combination*, and drawn his conclusions accordingly, than which, a more absurd and fatal mistake could not be made in any attempt to reason upon such a subject. It is quite true that oxygen in its pure state would be too stimulating for respiration; and hence, as we find it in the atmosphere, it is *diluted* with nitrogen—a gas wholly inert in its pure state. But it must be borne in mind that these gases are simply in a state of *mixture*, as composing the common air,—the nature of neither being essentially changed. Take another example: Oxygen and hydrogen, when merely *mixed*, still retain their gaseous forms and elementary properties, and constitute an exceedingly inflammable and explosive mixture; but when these two gases are *chemically* combined, the result is *water*—a substance wholly antagonistic in all its properties to those of either of its elements. Now it would be just as rational for one to contend, while drinking water, or breathing its vapor, that he was drinking or breathing oxygen or hydrogen, as to say that he was breathing oxygen, when it was chemically combined with any other substance. The universal law of chemistry is, that whenever any two substances are united by chemical affinity, the properties of both are changed, and the result is a third substance differing from either; and that the elements in such compounds can not act in their individual capacity till a positive decomposition is effected. And hence the perfect absurdity of supposing that we are breathing oxygen simply because we may be inhaling something containing oxygen as a chemical constituent. The resulting compound from a union of the most acrid substances is frequently inert. Common plaster of Paris is composed of sulphuric acid and lime; but when these two acrid substances are combined chemically, the result is that perfectly non-corrosive substance known as plaster of Paris.

These examples might be multiplied *ad infinitum*, but I shall offer but one other, which will not only illustrate remarkable changes wrought by chemical affinity between *different* substances, but where an equally surprising result is obtained *by combining the same substances, simply in "different proportions,"* and I can offer no better example than is seen in these very gases—oxygen and nitrogen in the different proportions in which they are capable of being united. Bearing in mind the nature and properties of these two gases, as simple substances, or when they are simply *mixed*, as in the atmosphere, let us see what changes are wrought by *chemically* combining them, and in different proportions.

These two gasses are capable of being combined in five different proportions:

1st. Oxygen 1, Nitrogen 1=Nitrous Oxide, [Laughing gas.]

2d. Oxygen 2, Nitrogen 1=Nitric Oxide.

3d. Oxygen 3, Nitrogen 1=Hypo-nitrous Acid.

4th. Oxygen 4, Nitrogen 1=Nitrous Acid.

5th. Oxygen 5, Nitrogen 1=Nitric Acid, [Aqua Fortis.]

It is not necessary to describe the peculiar qualities of all of these compounds. It is sufficient to say that while one proportion of oxygen, and one of nitrogen, *chemically combined*, forms the Exhilarating or Laughing Gas, *two* proportions of oxygen, with the same amount of nitrogen, forms the Nitric Oxide gas, a single inspiration of which would destroy life almost instantly. And *five proportions of oxygen*, with one proportion of nitrogen, constitutes Nitric Acid, or aqua fortis—a substance not tolerated by any part of the human system for a single moment.

But our learned champion for laughing gas gives us to understand that the more oxygen we combine with nitrogen, the more exhilarating and healthful it is. The air, containing but one proportion of the former, to three of the latter, he informs us, is not exhilarating; but by placing them on an equal footing in respect to quantity, the result is a most exhilarating and healthful atmosphere! But why stop here? Why, according to his logic, should not nitric acid be *five* times as exhilarating and healthy as nitrous oxide, or Laughing Gas?

The upshot of this whole matter is simply this: No man, however good a chemist he may be, can predict the nature of a compound, by any study of its elements,—much less its effect upon the human system. This is to be done, and only to be done by actual experiment. The chemist who first discovered that the combination of one equivalent of each of the two gases, oxygen and nitrogen, constitutes the exhilarating gas, was of course entirely familiar with the nature and properties of both of its constituents, and yet he was doubtless not a little surprised to find the resulting compound was of such a character, nor could his surprise have been less when he found that simply by doubling the amount of oxygen the compound was of a most deadly character as regards its effects upon the human system. There are now three substances in use as anæsthetic agents, to-wit: Sulphuric ether, chloroform and nitrous oxide—the chemical composition of each of which is as follows:

Nitrous oxide is composed of—Nitrogen 1 proportion, oxygen 1 proportion.

Sulphuric ether—Carbon 4 proportions, hydrogen 5 proportions, oxygen 1 proportion.

Chloroform—Carbon 2 proportions, Hydrogen 1 proportion, chlorine 3 proportions.

From the above it will be seen that inasmuch as the effect of each of these upon the human system is essentially the same, no judgment can be based upon their composition as to their nature in this relation. While the nitrous oxide is one half oxygen, ether contains but one-tenth, and chloroform not one particle of oxygen. The question now naturally arises, what is the effect of the nitrous oxide as shown by actual experiment? I answer in general terms of comparison.

*It is essentially the same as that produced by chloroform or ether*, with at least two drawbacks against it, which I shall hereafter point out. The first effect of any and all of these agents is to produce great and rapid excitement throughout the whole system—the blood-vessels are enlarged, and the blood circulates with nearly or quite double its usual rapidity. When this exhilarating stage has reached its hight, a stupor gradually supervenes, and the pulse correspondingly lessens, till frequently it becomes frightfully slow, and weak. Now it is very easy to account for this rapid exhilaration. A stimulant taken into the *stomach*, as ardent spirits, has to pass through the ordinary channels of digestion before it reaches the brain, but this, being absorbed by the blood, as it passes through the lungs, is taken almost instantly to the brain. But as to how it produces its anæsthetic effects, or entire insensibility to pain, is a question far more difficult of solution. There are two theories upon this point; the one accounts for the phenomenon by supposing that the extraordinary amount of blood carried to the brain produces actual compression upon this organ, and that the effect is the same as if the skull were actually depressed, which always produces insensibility.

Prof. Simpson, of Edinburgh, who first introduced chloroform into surgical and dental practice, accounts for this result by ascribing it to its peculiar effect upon the nervous system. He contends that those nerves which control motion are first affected by it, those of sensation next, and that it may under peculiar circumstances reach and paralize the vital nerves, in which case instant death must ensue. But it matters very little which theory we adopt, either in regard to its anæsthetic or fatal effects, as the modus operandi in producing them is essentially the same in either of the three; and as a legitimate conclusion, it follows that if there is in any given case, any good reason why ether or chloroform should not be used, there must be an equally strong one for avoiding the nitrous oxide gas. Neither of them if pure is, strictly speaking, poisonous, yet each of them will destroy life. My own view of these articles is this: that *any* agent capable of producing so unnatural a state, in so short a time, must necessarily thoroughly test the soundness of all who have this test applied, and if they have any physical ailments or defects, it will be quite sure to find them. It hardly need be added, that the employment of such agents should be confined to those thoroughly skilled in medical science, and at that, used with the utmost caution. In regard to the comparative effect of these three substances respectively, my conviction is, that while the nitrous oxide is by far the most exhilarating, its anæsthetic effects are greatly inferior to either of the others.

In whatever light we may view this "great and new discovery," (which, by the way, was made just 92 years ago), I have yet to see the smallest advantage, in any particular, it has over either chloroform or ether, and there are some objections to its employment as an anæsthetic agent, which do not pertain to either of the other articles. It is well known to almost every school-child that of the air we inhale, nearly eight per cent. is retained in the blood, and that a different and poisonous article, carbonic acid gas, is returned instead, and that oxygen is absolutely necessary to sustain life. Now in the inhalation of both chloroform or ether, a free supply of air is always taken into the lungs, in connection with the vapor of these substances. But how is it with the inhalation of nitrous oxide? This is breathed from a rubber bag (which is of course air-tight), and thus all the carbonic acid exhaled, is returned into the bag to be rebreathed. The gas must therefore necessarily soon be so contaminated as to become actually poisonous. Not only so, it is true that from the first, oxygen is positively excluded—for, as I have already demonstrated, oxygen, to be available for supporting life, must be free—that any amount of it in chemical combination with other substances, is not of the least account.

It therefore follows that in addition to every objection which can be urged against the use of chloroform or ether, these two must be superadded to nitrous oxide, to-wit: the patient is compelled to breathe more or less carbonic acid gas after the very first exhalation, and moreover an atmosphere totally deprived of the life-supporting principle—oxygen.

At best, no one could live longer, if confined to this gas, than he could live under water, or sustain life without oxygen, and this, as all know, would be but a few minutes But we are not dependant upon *theory* to exhibit the dangerous effects of this misnamed laughing gas. Facts are coming to us every day which not merely demonstrate its deleterious, but its *fatal* effects.

During the period in which I was in the habit of administering it in the Chemical Laboratory or Lecture Room, several serious cases, and one which came near to proving fatal, came under my own observation, and yet in no case did we intend to carry its effects beyond the exhilarating stage.

The last case above alluded to, was that of a young man apparently in full health, about twenty years of age, and in all respects seemed to be as good a subject as could be presented for a full dose. He had breathed it but a short time before he became entirely insensible, and apparently lifeless, and it was several hours before he could be fully restored to to consciousness, and several weeks before he fully recovered from its effects.

Prof. Amos Eaton, the founder of the Rensselaer Institute, uniformly in his lectures upon this subject, gave the strictest caution relative to its use, and related many instances of its deleterious effects even when prepared and administered by the most skillful and judicious hands.

In a letter just received from an intelligent dentist of a neighboring city, three cases of its bad effects are related, all occurring within the last two weeks. "In one of the cases the patient is still in such a state as to render his recovery doubtful." The lamentable and fatal case of Samuel P. Sears, who was a most estimable man of New York city, and who died within a few minutes after inhaling it, is fresh in the minds of us all.

In view of the nature of this gas, and of such facts as are above related, and many more of a like character doubtless might be collected, I appeal to those who are advertising it as perfectly harmless, to say whether they can conscientiously continue to do so. I have not written this article from any selfish motive, but simply and only from the hope that a candid exposition of the nature and character of this gas might be instrumental in saving some victims from its deleterious, or even fatal effects. A. WESTCOTT.

SYRACUSE, January, 1864.—*Syracuse Daily Journal.*

